# Advances in achieving lung and diaphragm-protective ventilation

**DOI:** 10.1097/MCC.0000000000001228

**Published:** 2024-11-14

**Authors:** Maarten J.W. van den Berg, Leo Heunks, Jonne Doorduin

**Affiliations:** Department of Intensive Care Medicine, Radboud University Medical Center, Nijmegen, The Netherlands

**Keywords:** diaphragm, interventions, lung, mechanical ventilation, monitoring

## Abstract

**Purpose of review:**

Mechanical ventilation may have adverse effects on diaphragm and lung function. Lung- and diaphragm-protective ventilation is an approach that challenges the clinician to facilitate physiological respiratory efforts, while maintaining minimal lung stress and strain. Here, we discuss the latest advances in monitoring and interventions to achieve lung- and diaphragm protective ventilation.

**Recent findings:**

Noninvasive ventilator maneuvers (P0.1, airway occlusion pressure, pressure-muscle index) can accurately detect low and excessive respiratory efforts and high lung stress. Additional monitoring techniques include esophageal manometry, ultrasound, electrical activity of the diaphragm, and electrical impedance tomography. Recent trials demonstrate that a systematic approach to titrating inspiratory support and sedation facilitates lung- and diaphragm protective ventilation. Titration of positive-end expiratory pressure and, if available, veno-venous extracorporeal membrane oxygenation sweep gas flow may further modulate neural respiratory drive and effort to facilitate lung- and diaphragm protective ventilation.

**Summary:**

Achieving lung- and diaphragm-protective ventilation may require more than a single intervention; it demands a comprehensive understanding of the (neuro)physiology of breathing and mechanical ventilation, along with the application of a series of interventions under close monitoring. We suggest a bedside-approach to achieve lung- and diaphragm protective ventilation targets.

## INTRODUCTION

**FIGURE 1:**
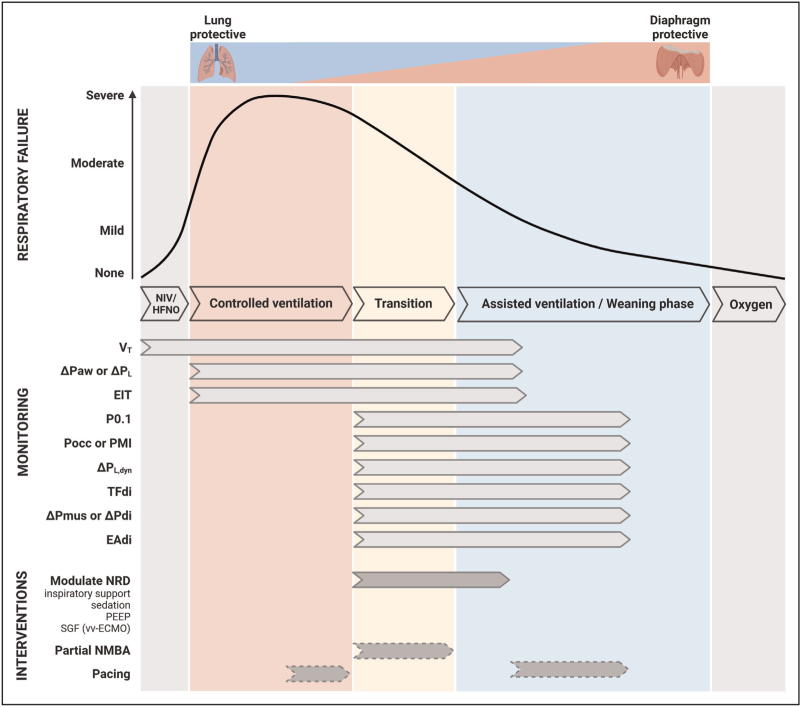
Monitoring parameters and interventions to achieve lung- and diaphragm-protective ventilation. Schematic representation of the monitoring parameters and interventions available to achieve lung- and diaphragm-protective ventilation in relation to the time course of respiratory failure and ventilatory support. In the early/acute phase of respiratory failure, lung-protective ventilation should be given priority, thereafter this priority gradually shifts to diaphragm-protective ventilation. Dashed arrows indicate experimental interventions that should not routinely be used in clinical practice. ΔPaw, airway driving pressure; ΔPdi, transdiaphragmatic pressure swing; ΔP_L_, static transpulmonary driving pressure; ΔP_L,dyn_, dynamic transpulmonary driving pressure; ΔPmus, respiratory muscle pressure swing; EAdi, electrical activity of the diaphragm; EIT, electrical impedance tomography; HFNO, high-flow nasal oxygen; NIV, noninvasive ventilation; NMBA, neuromuscular blocking agents; NRD, neural respiratory drive; PEEP, positive end-expiratory pressure; PMI, pressure muscle index; Pocc, airway occlusion pressure; SGF, sweep gas flow; TFdi, diaphragm thickening fraction; *V*_T_, tidal volume. vv-ECMO, veno-venous extracorporeal membrane oxygenation.

Mechanical ventilation is a lifesaving medical technology that is, however, associated with significant adverse effects. Lung- and diaphragm-protective ventilation is an approach that aims to limit the detrimental effects of mechanical ventilation on diaphragm function and minimize the risk for ventilator-induced lung injury. Implementation of this approach challenges the clinician to facilitate patient respiratory efforts that delicately balance between physiological levels of diaphragm activity and minimal lung stress and strain [1]. The application of lung- and diaphragm-protective ventilation is particularly challenging during the early transition from controlled ventilation to assisted ventilation, when spontaneous breathing efforts may be present (Fig. 1). 

**Box 1:**
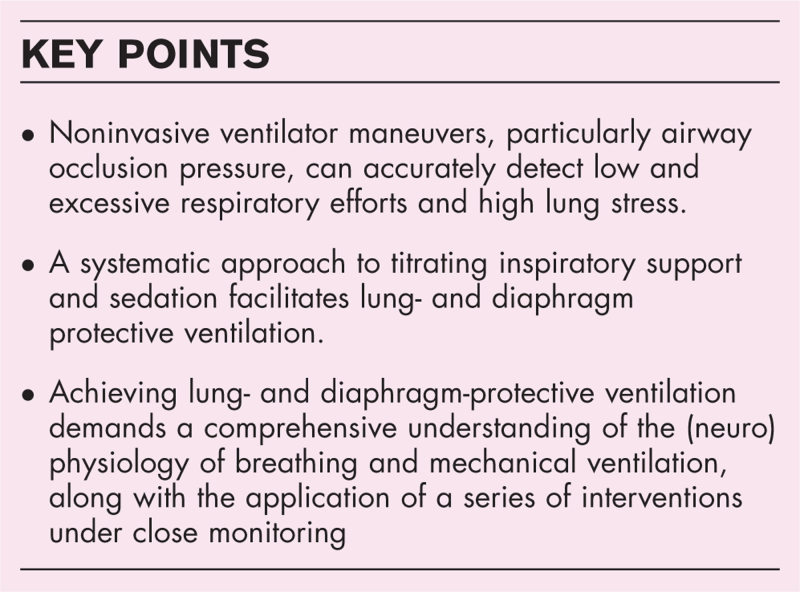
no caption available

In this review, after a brief overview of the mechanisms of lung and diaphragm injury associated with mechanical ventilation, we will discuss the latest advances in monitoring and interventions to facilitate lung- and diaphragm protective ventilation.

## MECHANISMS OF LUNG AND DIAPHRAGM INJURY

Ventilator-induced lung injury in mechanically ventilated patients is primarily driven by excessive lung stress and strain applied to an already compromised lung, often referred to as the ‘baby lung’. For lung tissue deformation (strain) to occur, a pressure (stress) must be applied. Larger deformations may result in alveolar overdistention and are generated by higher pressures (volutrauma/barotrauma). Excessive lung stress and strain can be induced by the mechanical ventilator, the patient's breathing effort, or a combination of both. Additionally, the repetitive opening and closing of alveoli with each breath generates shear stress and strain along the airway epithelium (atelectrauma). Finally, the induced mechanical injury triggers the release of pro-inflammatory cytokines (biotrauma) further compromising lung function.

Mechanical ventilation has been associated with diaphragm dysfunction. It should be acknowledged that in critically ill patients other factors, such as inflammation and certain drugs are associated with diaphragm dysfunction as well, and therefore we prefer the term critical illness associated diaphragm weakness instead of ventilator induced diaphragm dysfunction (VIDD). Ventilator over-assist, characterized by too high levels of inspiratory support, reduces or eliminates neural respiratory drive and consequently lowers diaphragmatic effort, leading to disuse atrophy [2,3]. Conversely, under-assistance forces the diaphragm to work excessively, possibly resulting in diaphragm injury, as demonstrated experimentally in both animal models and human studies [4,5]. Other possible causes of myotrauma include longitudinal atrophy resulting from high PEEP and also eccentric contractions – lengthening of the muscle during contraction – resulting from patient-ventilator dyssynchrony [6,7]. However, the clinical significance of these last two mechanisms remains to be established.

The mechanisms of lung and diaphragm injury in mechanically ventilated patients are discussed in detail elsewhere [8–10].

## ADVANCES IN MONITORING

Parameters that reflect respiratory effort as well as lung stress and strain can be monitored to achieve lung- and diaphragm-protective ventilation. Here, we will describe different parameters that can be obtained directly from the ventilator or through additional monitoring techniques. Table 1 provides an overview of the parameters along with their proposed target values.

**Table 1:** Lung- and diaphragm-protective targets

Parameter	Calculation/measurement	Monitoring goal and applicability	Target value
Tidal volume (*V*_*T*_)	Directly from ventilator	Available on every ventilator to estimate risk of lung injury.	4–8 ml/kg PBW
P0.1	Inspiratory drop Paw in first 100 ms during an expiratory hold.	Available on every ventilator. Maneuver to estimate neural respiratory drive, but inferior to Pocc for respiratory effort.	1–4 cmH_2_O
Occlusion pressure (Pocc)	Inspiratory drop in Paw during an expiratory hold.	Ventilator maneuver to estimate respiratory effort and lung stress, recommended for monitoring majority of patients.	7–15 cmH_2_O
Pressure muscle index (PMI)	Pplat – Ppeak; Pplat measured during inspiratory hold under assisted ventilation.	Ventilator maneuver to estimate respiratory effort. Challenging because it requires a stable plateau pressure.^a^	<6 cmH_2_O
Static airway driving pressure (ΔPaw)	Pplat – PEEPtot; measured during an inspiratory and expiratory hold, respectively.	Easy ventilator maneuver, recommended for monitoring global lung stress and strain during controlled ventilation. During assisted ventilation its usefulness as a surrogate of tidal lung stress is limited due to inspiratory (and expiratory) muscle activity.	<15 cmH_2_O
Static transpulmonary driving pressure (ΔP_L_)	P_L_ end-inspiratory – P_L_end-expiratory; measured during an inspiratory and expiratory hold, respectively. (P_L_ = Paw – Pes).	Requires an esophageal balloon catheter, recommended in selected cases to accurately monitoring lung stress and strain.	<12 cmH_2_O
Dynamic transpulmonary pressure (ΔP_L,dyn_)	Swing in ΔP_L_ during inspiration (P_L_ = Paw – Pes)	Requires an esophageal balloon catheter, recommended in selected cases to accurately monitoring respiratory effort and dynamic lung stress	<20 cmH_2_O
Predicted ΔP_L,dyn_	Pinsp – 2/3 x Pocc.	Can be used to detect high respiratory effort and dynamic lung stress.	<22 cmH_2_O
Thickening fraction (TFdi)	((Tdi_ei_ – Tdi_ee_) / Tdi_ee_) x 100	Requires expertise in diaphragm ultrasound. Not a first choice to monitor respiratory effort.	15–40%
Respiratory muscle pressure (ΔPmus)	Pmus = Pcw,rel – Pes	Gold standard to monitor respiratory muscle effort, but requires an esophageal balloon catheter. Recommended in selected cases.	3–15 cmH_2_O
Transdiaphragmatic pressure swing (ΔPdi)	Pdi = Pgas – Pes	Gold standard to monitor diaphragm effort, but requires a gastric and esophageal balloon catheter. Recommended in selected cases.	3–12 cmH_2_O
Electrical activity of the diaphragm (EAdi)	Directly from ventilator	Gold standard to monitor neural respiratory drive, but requires a dedicated catheter and ventilator.	Not available

Note that the target values reflect general consensus. Paw, airway pressure; PBW, predicted bodyweight; Pcw,rel, chestwall elastic recoil pressure; PEEPtot, total positive end-expiratory pressure; Pes, esophageal pressure; Pga, gastric pressure; Pinsp, inspiratory pressure set on the ventilator; Ppeak, peak airway pressure; Pplat, plateau airway pressure; Tdi_ee_, end-expiratory diaphragm thickness; Tdi_ei_, end-inspiratory diaphragm thickness.

a Pplat can be measured on assisted ventilation when the three criteria of a readable Pplat are met during an inspiratory hold.

### Monitoring neural respiratory drive or respiratory effort

Neural respiratory drive is defined as the neural output of the respiratory centers located in the brainstem. Respiratory drive results in activation of the respiratory pump (effort), but the relationship between respiratory drive and effort is complex [11].

At the bedside, neural respiratory drive and respiratory effort can be estimated using parameters directly obtained from ventilator waveforms, including P0.1 and Pocc. The occluded end-expiratory airway pressure (Pocc) is the drop in airway pressure during an inspiration against an occluded airway, while the P0.1 is defined as the drop in airway pressure in the first 100 ms of an occluded inspiration. These measures have been used to detect low and excessive respiratory muscle pressure (Pmus) and dynamic transpulmonary pressure (ΔP_L,dyn_) [12,13,14^▪▪^]. Recent work demonstrated that Pocc outperforms P0.1 in detecting patients with high diaphragmatic effort [14^▪▪^].

During assisted ventilation, when the inspiratory muscles relax during an end-inspiratory hold, the plateau pressure (Pplat) can exceed the peak pressure (Ppeak). The difference between Pplat and Ppeak is referred to as the pressure muscle index (PMI), which has been reported as a measure of patient effort during assisted mechanical ventilation. Recently, PMI has been shown to reliably predict high and low respiratory effort during assisted ventilation [15]. For accurate measurement of Pplat during an inspiratory hold, three criteria must be fulfilled: time to reach plateau <800 ms; total duration of the plateau >2 s; and stable plateau (variation < 0.6 cmH_2_O) [16].

Ultrasound can be used to quantify inspiratory thickening of the diaphragm. Thickening fraction of the diaphragm (TFdi) may serve as a surrogate for respiratory effort, and thereby facilitate identification of patients with low diaphragm activity (over-assistance). However, the relationship between TFdi and diaphragm contractility remains a subject of debate [17], and there is currently no evidence to suggest that TFdi provides additional value over Pocc as a measure of respiratory effort. Additionally, diaphragm ultrasound does not allow for continuous monitoring.

Using esophageal manometry, swings in esophageal pressure (ΔPes) can be measured to estimate the pressure generated by the respiratory muscles (ΔPmus). ΔPmus is calculated as the difference between Pes and the chest wall recoil pressure (Pcw,rel) at a given V_T_. The specific inspiratory effort of the diaphragm can be quantified using transdiaphragmatic pressure (ΔPdi), which is calculated as the difference between gastric pressure (Pga) and Pes. An increase in Pga during expiration indicates expiratory muscle activity.

Electrical activity of the diaphragm (EAdi) reflects the neural respiratory drive rather than the force generated by the diaphragm. EAdi can be recorded using a dedicated nasogastric catheter equipped with multiple electrodes. Due to marked interindividual variability in the signal, no specific target value for EAdi have been be established [18]. Nevertheless, EAdi is particularly useful to detect over-assistance and poor patient-ventilator interaction[19].A limitation of this technique is that it requires a dedicated ventilator.

### Monitoring lung strain and stress

Maintaining a low tidal volume (*V*_T_) minimizes the risk of lung injury by preventing alveolar overdistension. However, the use of *V*_T_ as a measure of lung strain and stress is limited. To estimate lung strain, *V*_T_ is divided by end-expiratory lung volume, which is not easily accessible. Inspiratory and expiratory hold maneuvers on the ventilator can be used to approximate global lung stress by correcting *V*_T_ for the compliance of the respiratory system (Crs), known as the airway driving pressure (ΔPaw = plateau pressure − total PEEP). It is important to note that ΔPaw reflects stress on the entire respiratory system, including the lungs and chest wall. Esophageal pressure measurements allow to assess lung stress, that is the transpulmonary pressure driving pressure (ΔP_L_). ΔP_L_ can be measured under static (zero-flow) or dynamic conditions. ΔP_L,dyn_ may overestimate lung stress due to the resistance component.

In patients under assisted ventilation, it is important to recognize that Pplat and total PEEP (PEEP_tot_) can be influenced by respiratory muscle activity. In the presence of expiratory muscle activity, ΔPaw underestimates ΔP_L_[20]. This phenomenon limits the usefulness of ΔPaw as a surrogate of tidal lung stress in patients with active breathing. Airway occlusion maneuvers may provide a noninvasive estimate of lung stress. Although Pocc and P0.1 are unable to predict exact lung stress values, both can identify patients with high lung stress [14^▪▪^]. Note that Pocc outperforms P0.1. Additionally, both Pocc and P0.1 are moderately correlated with mechanical power [14^▪▪^].

Electrical impedance tomography (EIT) allows real-time imaging of ventilation and lung perfusion. EIT uses electrodes placed circumferentially around the thorax. EIT is mainly used to assess regional lung collapse or overdistention. [21] In spontaneously breathing patients, EIT can quantify pendelluft, where regional overdistention may occur despite achieving a safe *V*_T_. However, a significant limitation of EIT is the lack of evidence for target values or the optimal balance between collapse and overdistention.

## ADVANCES IN INTERVENTIONS

Here, we will discuss the recent advances in interventions to achieve lung- and diaphragm protective ventilation. Figure 1 presents an overview of these interventions in relation to the time course of ICU admission for a patient with acute respiratory failure.

### Modulation of neural respiratory drive

In the early phase of critical illness, reducing sedation level and inspiratory support to facilitate the transition from controlled ventilation to assisted ventilation often results in excessive respiratory efforts [22^▪^,23^▪^]. Therefore, modulation of neural respiratory drive, and subsequent modulation of respiratory effort, is a key strategy to mitigate the development of lung and diaphragm injury.

Titrating inspiratory support and sedation should be considered as a first step to modulate neural respiratory drive. To date, two physiological randomized controlled trials studied the effect of inspiratory support titration [22^▪^,23^▪^]. These trials demonstrate that a systematic approach to titrating inspiratory support (and sedation) facilitates ventilation within predefined targets of lung- and diaphragm protective ventilation. However, the short duration of these physiological trials (≤24 h) does not allow conclusions on long-term feasibility and effects on patient-centered outcomes. Furthermore, lung- and diaphragm-protective targets were not achieved in all patients, underscoring the necessity for additional interventions.

The effects of PEEP on modulating neural respiratory drive and respiratory effort are not straightforward: higher PEEP may either increase or decrease respiratory effort via different mechanisms. First, it appears that respiratory effort decreases if higher PEEP yields increased lung compliance (alveolar recruitment), whereas respiratory effort increases if lung compliance decreases (alveolar overinflation) [23^▪^,24^▪^]. Second, PEEP increases end-expiratory volume and subsequently leads to flattening of the diaphragm [25]. As a result, diaphragm fibers may be displaced from their optimal length-tension relationship, thereby reducing diaphragm contractile efficiency [26,27]. Third, experimental data suggests that PEEP induces diaphragm remodeling through longitudinal atrophy and that low PEEP may increase the level of (injurious) eccentric diaphragm contractions [6]. However, the latter two mechanisms have not yet been confirmed in vivo. A recent review on the role of PEEP in diaphragm-protective ventilation discusses these mechanism in more detail [28]. Overall, recent findings confirm that modifications in PEEP affect neural respiratory drive and respiratory effort. Nevertheless, the complex interplay between multiple mechanisms renders the effect of PEEP unpredictable in clinical practice. Therefore, PEEP as an intervention to achieve lung- and diaphragm-protective ventilation should be carefully titrated to the individual patient.

Carbon dioxide is a major determinant of neural respiratory drive. Therefore, extracorporeal CO_2_ removal reduces ventilatory demands and can attenuate neural respiratory drive in spontaneously breathing patients [29–31]. In a physiological randomized controlled trial, increasing sweep gas flow (facilitating extracorporeal CO_2_ removal), after optimizing inspiratory support and sedation, increases the likelihood of facilitating lung- and diaphragm-protective ventilation [23^▪^]. Recently, results of an in-silico trial supported the adjunctive role of extracorporeal CO_2_ removal to attenuate neural respiratory drive, while extracorporeal CO_2_ removal alone had a very low rate of success [32]. Future trials are required to establish the best strategy for extracorporeal CO_2_ removal to achieve lung- and diaphragm-protective targets in the transition from controlled to assisted mechanical ventilation in patients with excessive respiratory efforts.

### Neuromechanical uncoupling of the diaphragm

An experimental strategy to diminish strenuous respiratory efforts while maintaining diaphragm activity is pharmacologically induced neuromechanical uncoupling of the diaphragm. Initially, this strategy was proposed by inducing partial neuromuscular blockade using low doses of rocuronium [33]. More recently, it was demonstrated that partial neuromuscular blockade was well tolerated and effectively attenuated respiratory effort when it was refractory to other interventions [23^▪^]. An alternative approach is perineural administration of lidocaine to the phrenic nerve, i.e. a phrenic nerve block [34,35]. In a proof-of-concept study in nine patients, it was found that bilateral phrenic nerve block reduced V, peak transpulmonary pressure and ΔP_L_ in the short term [34].

Importantly, pharmacologically induced neuromechanical uncoupling of the diaphragm does not reduce neural respiratory drive, but constraints the capacity of the diaphragm to generate pressure. In fact, neural respiratory drive is most likely increased in these patients, which may contribute to dyspnea sensation. Furthermore, the feasibility and safety of maintaining this intervention over hours to days remains to be established.

### Diaphragm neurostimulation

Diaphragm neurostimulation is an emerging technique that involves application of electrical or magnetic stimuli directly to the phrenic nerves or in their close vicinity to elicit diaphragm contractions. A complete overview and comparison of techniques can be found elsewhere [36,37]. The main rational for diaphragm neurostimulation is to prevent disuse atrophy during controlled mechanical ventilation or increase diaphragm strength in difficult-to-wean patients. Additionally, it might reduce lung stress and strain by recruiting more posterior lung regions compared to mechanical ventilation alone. Proof-of-concept and feasibility studies, along with initial randomized controlled trials in critically ill patients, have yielded promising results, indicating the potential for diaphragm neurostimulation [38–43]. Nevertheless, additional data are necessary to validate its long-term feasibility and, more importantly, to ascertain its effect on diaphragm function, lung stress and strain, and clinically relevant outcome parameters.

## BEDSIDE APPROACH TO LUNG- AND DIAPHRAGM-PROTECTIVE VENTILATION

Given the recent findings discussed above, we suggest a bedside-approach to achieve lung- and diaphragm protective ventilation targets in patients beyond the acute phase of critical illness.

During controlled ventilation, we propose that monitoring of lung stress and strain can be achieved by measuring airway driving pressure and *V*_T_. Measurement of transpulmonary driving pressure using an esophageal catheter is recommended, if available and clinicians are sufficiently trained, in selected cases. For example, patients with very severe oxygenation impairment, obesity or abnormal thoracic cage. Using EIT, collapse or overdistention of lung regions may provide additional insight into the effect of ventilator settings on lung stress and strain. During the transition phase and during assisted ventilation, predicted dynamic transpulmonary pressure and *V*_T_ can be used to identify excessive dynamic lung stress and strain. If so, measurement of transpulmonary driving pressure using an esophageal catheter is recommended. In patients with active breathing, we propose monitoring of respiratory effort with the end-expiratory airway occlusion (Pocc) maneuver. If available, measurement of esophageal or transdiaphragmatic pressure is recommended in selected cases, such as activation of expiratory muscles or discrepancy between Pocc and clinical evaluation of respiratory effort. See Table 1 for target values.

**FIGURE 2:**
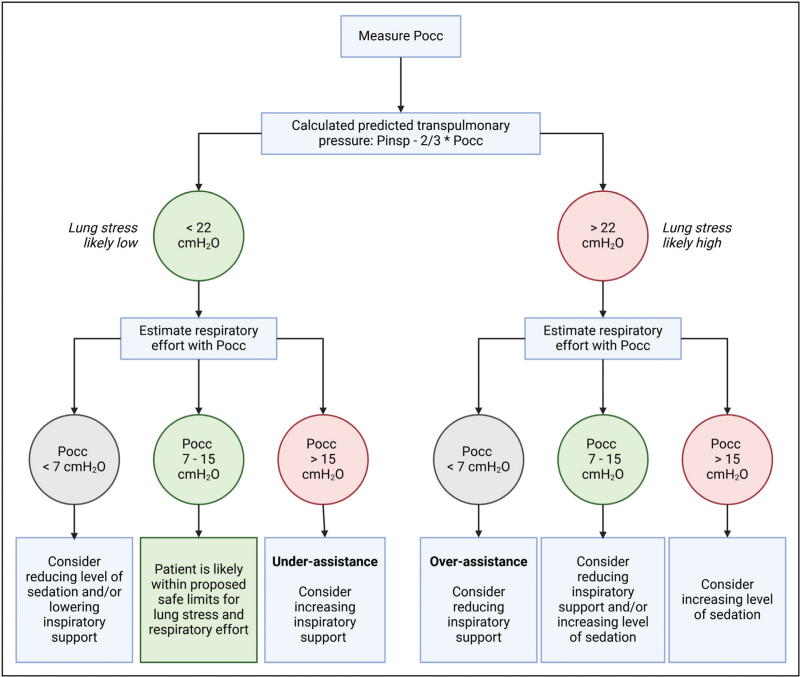
Bedside approach to achieve lung- and diaphragm-protective ventilation based on the occluded inspiratory airway pressure. In this approach the occluded inspiratory airway pressure (Pocc) is measured to estimate both lung stress and diaphragm effort. Pinsp is the inspiratory pressure set on the ventilator without including the rise in pressure that can sometimes be observed during the latter part of inspiration. The protocol can be repeated hourly, or when patient breathing effort has likely changed such as after changing ventilator settings. Note that prospective studies are required to assess whether using this protocol results in lung stress and effort in purported safe ranges. Figure adapted from [14^▪▪^].

To facilitate the transition from controlled mechanical ventilation to an assisted mode, sedation and respiratory rate should be reduced under close monitoring. We propose the following protocol based on the occluded inspiratory airway pressure to achieve values of lung stress and respiratory effort within a protective range during assisted ventilation (Fig. 2). First, calculate the predicted ΔP_L,dyn_ based on Pocc to detect high lung stress. Patients with a predicted P_L,dyn_ <22 cmH_2_O have a purported safe P_L,dyn_ (<20 cmH_2_O) in 86% of the cases [14^▪▪^]. Second, Pocc without conversion is used to assess whether patients likely have transdiaphragmatic pressure in the purported safe range (3 - 12 cmH_2_O). Patient with a Pocc between 7 and 15 cmH_2_O have purported safe diaphragm effort in 89% of the cases [14^▪▪^]. Third, when either lung stress is too high or respiratory effort is outside the safe range, consider adjusting sedation and/or inspiratory support. The step size of the change in inspiratory support may depend on its observed effect, but we recommend starting with steps of 2 cmH_2_O. Given that sedation scores are poorly correlated with respiratory drive [44], we cannot recommend specific sedation targets. The level of sedation required is determined by a weighted balance of respiratory drive, patient comfort, and the potential complications associated with deep and prolonged sedation. The protocol can be repeated hourly, or when patient breathing effort has likely changed such as after changing ventilator settings. When titration of inspiratory support and sedation fails to achieve lung- and diaphragm protective ventilation, PEEP can be titrated in an attempt to reduce or increase respiratory effort. Although lung protection with PEEP should be prioritized above diaphragm protection. If patients are on ECMO, sweep gas flow can be adjusted to modulate respiratory effort.

Currently, we do not recommend routine clinical application of partial neuromuscular blockade, phrenic nerve blocking or neurostimulation of the diaphragm to achieve lung- and diaphragm protective ventilation targets. Future studies should first demonstrate the effectiveness and safety on the long-term for these strategies.

## CONCLUSION

To conclude, achieving lung- and diaphragm-protective ventilation necessitates more than a single adjustment in ventilator settings or a single pharmacological intervention; it demands a comprehensive understanding of the (neuro)physiology of breathing and mechanical ventilation, along with the application of a series of interventions under close monitoring.

## Acknowledgements

*None.*

### Financial support and sponsorship

*None.*

### Conflicts of interest

*L.H. has received consulting fees from Liberate Medical (USA) and Pulmotech (NL). L.H. has received a speaker/travel fee from Mindray. J.D. has received a consulting fee from Springer (NL). For the remaining authors none were declared.*

## REFERENCES AND RECOMMENDED READING

Papers of particular interest, published within the annual period of review, have been highlighted as:

▪ of special interest
▪▪ of outstanding interest

## References

[1] Goligher EC Dres M Patel BK. Lung- and diaphragm-protective ventilation. Am J Respir Crit Care Med 2020; 202:950–961. 32516052 10.1164/rccm.202003-0655CP PMC7710325

[2] Levine S Nguyen T Taylor N. Rapid disuse atrophy of diaphragm fibers in mechanically ventilated humans. N Engl J Med 2008; 358:1327–1335. 18367735 10.1056/NEJMoa070447

[3] Hooijman PE Beishuizen A Witt CC. Diaphragm muscle fiber weakness and ubiquitin-proteasome activation in critically ill patients. Am J Respir Crit Care Med 2015; 191:1126–1138. 25760684 10.1164/rccm.201412-2214OC PMC4451621

[4] Jiang TX Reid WD Belcastro A Road JD. Load dependence of secondary diaphragm inflammation and injury after acute inspiratory loading. Am J Respir Crit Care Med 1998; 157:230–236. 9445304 10.1164/ajrccm.157.1.9702051

[5] Gea J Zhu E Galdiz JB. Functional consequences of eccentric contractions of the diaphragm. Arch Bronconeumol 2009; 45:68–74. 19232267 10.1016/j.arbres.2008.04.003

[6] Lindqvist J van den Berg M van der Pijl R. Positive end-expiratory pressure ventilation induces longitudinal atrophy in diaphragm fibers. Am J Respir Crit Care Med 2018; 198:472–485. 29578749 10.1164/rccm.201709-1917OC PMC6118031

[7] de Vries HJ Jonkman AH Holleboom MC. Diaphragm activity during expiration in ventilated critically ill patients. Am J Respir Crit Care Med 2024; 209:881–883. 38190708 10.1164/rccm.202310-1845LE

[8] Dres M Goligher EC Heunks LMA Brochard LJ. Critical illness-associated diaphragm weakness. Intensive Care Med 2017; 43:1441–1452. 28917004 10.1007/s00134-017-4928-4

[9] Slutsky AS Ranieri VM. Ventilator-induced lung injury. N Engl J Med 2013; 369:2126–2136. 24283226 10.1056/NEJMra1208707

[10] Brochard L Slutsky A Pesenti A. Mechanical ventilation to minimize progression of lung injury in acute respiratory failure. Am J Respir Crit Care Med 2017; 195:438–442. 27626833 10.1164/rccm.201605-1081CP

[11] Jonkman AH de Vries HJ Heunks LMA. Physiology of the respiratory drive in ICU patients: implications for diagnosis and treatment. Crit Care 2020; 24:104. 32204710 10.1186/s13054-020-2776-z PMC7092542

[12] Bertoni M Telias I Urner M. A novel noninvasive method to detect excessively high respiratory effort and dynamic transpulmonary driving pressure during mechanical ventilation. Crit Care 2019; 23:346. 31694692 10.1186/s13054-019-2617-0 PMC6836358

[13] Yang YL Liu Y Gao R. Use of airway pressure-based indices to detect high and low inspiratory effort during pressure support ventilation: a diagnostic accuracy study. Ann Intensive Care 2023; 13:111. 37955842 10.1186/s13613-023-01209-7 PMC10643759

[14▪▪] de Vries HJ Tuinman PR Jonkman AH. Performance of noninvasive airway occlusion maneuvers to assess lung stress and diaphragm effort in mechanically ventilated critically ill patients. Anesthesiology 2023; 138:274–288. 36520507 10.1097/ALN.0000000000004467

[15] Gao R Zhou JX Yang YL. Use of pressure muscle index to predict the contribution of patient's inspiratory effort during pressure support ventilation: a prospective physiological study. Front Med 2024; 11:1390878. 10.3389/fmed.2024.1390878 PMC11082330 38737762

[16] Bianchi I Grassi A Pham T. Reliability of plateau pressure during patient-triggered assisted ventilation. Analysis of a multicentre database. J Crit Care 2022; 68:96–103. 34952477 10.1016/j.jcrc.2021.12.002

[17] Poulard T Bachasson D Fosse Q. Poor correlation between diaphragm thickening fraction and transdiaphragmatic pressure in mechanically ventilated patients and healthy subjects. Anesthesiology 2022; 136:162–175. 34788380 10.1097/ALN.0000000000004042

[18] Bertoni M Spadaro S Goligher EC. Monitoring patient respiratory effort during mechanical ventilation: lung and diaphragm-protective ventilation. Crit Care 2020; 24:106. 32204729 10.1186/s13054-020-2777-y PMC7092676

[19] Piquilloud L Beloncle F Richard JM. Information conveyed by electrical diaphragmatic activity during unstressed, stressed and assisted spontaneous breathing: a physiological study. Ann Intensive Care 2019; 9:89. 31414251 10.1186/s13613-019-0564-1 PMC6692797

[20] Stamatopoulou V Akoumianaki E Vaporidi K. Driving pressure of respiratory system and lung stress in mechanically ventilated patients with active breathing. Crit Care 2024; 28:19. 38217038 10.1186/s13054-024-04797-3 PMC10785492

[21] Pavlovsky B Desprez C Richard JC. Bedside personalized methods based on electrical impedance tomography or respiratory mechanics to set PEEP in ARDS and recruitment-to-inflation ratio: a physiologic study. Ann Intensive Care 2024; 14:1. 38180544 10.1186/s13613-023-01228-4 PMC10769993

[22▪] de Vries HJ Jonkman AH de Grooth HJ. Lung- and diaphragm-protective ventilation by titrating inspiratory support to diaphragm effort: a randomized clinical trial. Crit Care Med 2022; 50:192–203. 35100192 10.1097/CCM.0000000000005395 PMC8797006

[23▪] Dianti J Fard S Wong J. Strategies for lung- and diaphragm-protective ventilation in acute hypoxemic respiratory failure: a physiological trial. Crit Care 2022; 26:259. 36038890 10.1186/s13054-022-04123-9 PMC9422941

[24▪] Bello G Giammatteo V Bisanti A. High vs low PEEP in patients with ARDS exhibiting intense inspiratory effort during assisted ventilation: a randomized crossover trial. Chest 2024; 165:1392–1405. 38295949 10.1016/j.chest.2024.01.040

[25] Formenti P Miori S Galimberti A Umbrello M. The effects of positive end expiratory pressure and lung volume on diaphragm thickness and thickening. Diagnostics (Basel) 2023; 13:1157. 36980465 10.3390/diagnostics13061157 PMC10047794

[26] Jansen D Jonkman AH Vries HJ. Positive end-expiratory pressure affects geometry and function of the human diaphragm. J Appl Physiol 2021; 131:1328–1339. 34473571 10.1152/japplphysiol.00184.2021

[27] Widing H Pellegrini M Chiodaroli E. Positive end-expiratory pressure limits inspiratory effort through modulation of the effort-to-drive ratio: an experimental crossover study. Intensive Care Med Exp 2024; 12:10. 38311676 10.1186/s40635-024-00597-9 PMC10838888

[28] Wennen M Claassen W Heunks L. Setting positive end-expiratory pressure: role in diaphragm-protective ventilation. Curr Opin Crit Care 2024; 30:61–68. 38085880 10.1097/MCC.0000000000001126

[29] Jung C Gillmann HJ Stueber T. Modification of respiratory drive and lung stress by level of support pressure and ECMO sweep gas flow in patients with severe COVID-19-associated acute respiratory distress syndrome: an exploratory retrospective analysis. J Cardiothorac Vasc Anesth 2024; 38:221–229. 38197786 10.1053/j.jvca.2023.09.040

[30] Mauri T Grasselli G Suriano G. Control of respiratory drive and effort in extracorporeal membrane oxygenation patients recovering from severe acute respiratory distress syndrome. Anesthesiology 2016; 125:159–167. 26999639 10.1097/ALN.0000000000001103

[31] Crotti S Bottino N Ruggeri GM. Spontaneous breathing during extracorporeal membrane oxygenation in acute respiratory failure. Anesthesiology 2017; 126:678–687. 28212205 10.1097/ALN.0000000000001546

[32] Ratano D Zhang B Dianti J. Lung- and diaphragm-protective strategies in acute respiratory failure: an in silico trial. Intensive Care Med Exp 2024; 12:20. 38416269 10.1186/s40635-024-00606-x PMC10902250

[33] Doorduin J Nollet JL Roesthuis LH. Partial neuromuscular blockade during partial ventilatory support in sedated patients with high tidal volumes. Am J Respir Crit Care Med 2017; 195:1033–1042. 27748627 10.1164/rccm.201605-1016OC

[34] Pereira SM Sinedino BE Costa ELV. Phrenic nerve block and respiratory effort in pigs and critically ill patients with acute lung injury. Anesthesiology 2022; 136:763–778. 35348581 10.1097/ALN.0000000000004161

[35] Levis A Gardill M Bachmann KF. Bilateral phrenic nerve block to reduce hazardous respiratory drive in a mechanically ventilated patient with COVID-19-A case report. Clin Case Rep 2024; 12:e8850. 38721551 10.1002/ccr3.8850 PMC11077186

[36] Etienne H Morris IS Hermans G. Diaphragm Neurostimulation Assisted Ventilation in Critically Ill Patients. Am J Respir Crit Care Med 2023; 207:1275–1282. 36917765 10.1164/rccm.202212-2252CP PMC10595441

[37] Panelli A Verfuss MA Dres M. Phrenic nerve stimulation to prevent diaphragmatic dysfunction and ventilator-induced lung injury. Intensive Care Med Exp 2023; 11:94. 38109016 10.1186/s40635-023-00577-5 PMC10728426

[38] Morris IS Bassi T Bellissimo CA. Proof of concept for continuous on-demand phrenic nerve stimulation to prevent diaphragm disuse during mechanical ventilation (STIMULUS): a phase 1 clinical trial. Am J Respir Crit Care Med 2023; 208:992–995. 37642635 10.1164/rccm.202305-0791LE

[39] Dres M de Abreu MG Merdji H. Randomized clinical study of temporary transvenous phrenic nerve stimulation in difficult-to-wean patients. Am J Respir Crit Care Med 2022; 205:1169–1178. 35108175 10.1164/rccm.202107-1709OC PMC9872796

[40] Mueller G Aszalos E Krause S. Safety and feasibility of noninvasive electromagnetic stimulation of the phrenic nerves. Respir Care 2023; 68:602–610. 36878642 10.4187/respcare.10568 PMC10171341

[41] Panelli A Bartels HG Krause S. First noninvasive magnetic phrenic nerve and diaphragm stimulation in anaesthetized patients: a proof-of-concept study. Intensive Care Med Exp 2023; 11:20. 37081235 10.1186/s40635-023-00506-6 PMC10118662

[42] Panelli A Grimm AM Krause S. Noninvasive electromagnetic phrenic nerve stimulation in critically ill patients: a feasibility study. Chest 2024; 166:502–510. 38403186 10.1016/j.chest.2024.02.035 PMC11443241

[43] Parfait M Rohrs E Joussellin V. An initial investigation of diaphragm neurostimulation in patients with acute respiratory distress syndrome. Anesthesiology 2024; 140:483–494. 38088791 10.1097/ALN.0000000000004873

[44] Dzierba AL Khalil AM Derry KL. Discordance between respiratory drive and sedation depth in critically ill patients receiving mechanical ventilation. Crit Care Med 2021; 49:2090–2101. 34115638 10.1097/CCM.0000000000005113 PMC8602777

